# Time of delivery among low‐risk women at 37–42 weeks of gestation and risks of stillbirth and infant mortality, and long‐term neurological morbidity

**DOI:** 10.1111/ppe.12868

**Published:** 2022-03-04

**Authors:** Neda Razaz, Giulia M. Muraca, Katharina Fink, Amélie Boutin, Sid John, Sarka Lisonkova, Olof Stephansson, Sven Cnattingius, K. S. Joseph

**Affiliations:** ^1^ Clinical Epidemiology Division Department of Medicine Solna Karolinska Institutet Stockholm Sweden; ^2^ Department of Obstetrics and Gynaecology BC Children’s and Women’s Hospital and Health Centre and the University of British Columbia Vancouver BC Canada; ^3^ Department of Clinical Neuroscience Karolinska Institutet Stockholm Sweden; ^4^ Centrum for Neurology Academical Specialist Center Stockholm Sweden; ^5^ School of Population and Public Health University of British Columbia Vancouver BC Canada; ^6^ Department of Women’s Health Karolinska University Hospital Stockholm Sweden

**Keywords:** gestational age, infant mortality, neurodevelopmental disorder, stillbirth, timing of delivery

## Abstract

**Background:**

The most important knowledge gap in connection with obstetric management for time of delivery in term low‐risk pregnancies relates to the absence of information on long‐term neurodevelopmental outcomes.

**Objectives:**

We examined risks of stillbirth, infant mortality, cerebral palsy (CP) and epilepsy among low‐risk pregnancies.

**Methods:**

In this population‐based Swedish study, we identified, from 1998 to 2019, 1,773,269 singleton infants born between 37 and 42 completed weeks in women with low‐risk pregnancies. Poisson log‐linear regression models were used to examine the association between gestational age at delivery and stillbirth, infant mortality, CP and epilepsy. Adjusted rate ratios (RR) and 95% confidence intervals expressing the effect of birth at a particular gestational week compared with birth at a later gestational week were estimated.

**Results:**

Compared with those born at a later gestation, RRs for stillbirth and infant mortality were higher among births at 37 weeks' and 38 weeks' gestation. The RRs for infant mortality were approximately 20% and 25% lower among births at 40 or 41 weeks compared with those born at later gestation, respectively. Infants born at 37 and 38 weeks also had higher RRs for CP (vs infants born at ≥38 and ≥39 weeks, respectively), while those born at 39 gestation had similar RRs (vs infants born at ≥40 weeks); infants born at 40 and 41 weeks had lower RRs of CP (vs those born at ≥41 and 42 weeks, respectively). The RRs for epilepsy were higher in those born at 37 and 38 weeks compared with those born at later gestation.

**Conclusions:**

Among low‐risk pregnancies, birth at 37 or 38 completed weeks' gestation is associated with increased risks of stillbirth, infant mortality and neurological morbidity, while birth at 39–40 completed weeks is associated with reduced risks compared with births at later gestation.

**Synopsis**

**Study question**

What is the impact of time of delivery in low‐risk pregnancies at ≥37 weeks' gestation on risks of stillbirth, infant mortality and neurological disorders in offspring?

**What is already known**

The time of delivery in low‐risk pregnancies is of particular concern as the clinician must balance the higher risks of respiratory morbidity and other neonatal complications associated with early‐term delivery with the risks of post‐maturity. There is a critical knowledge gap concerning the time of delivery in low‐risk women at ≥37 weeks' gestation and long‐term outcomes.

**What the study adds**

Being born at 39 and 40 weeks reduces risks of stillbirth, infant mortality and cerebral palsy, indicating that 39+0 to 40+6 weeks is the optimum time for delivery for low‐risk pregnancies.

## 1 BACKGROUND

Increasing rates of obstetric intervention have led to a left shift in the gestational age distribution, with an increased rate of early‐term births and a decreased rate of post‐term births.^1^, ^2^ However, in prolonged but otherwise uncomplicated pregnancies, active versus conservative expectant management is still under debate.^3^ Proponents of earlier‐term delivery argue that the ability of the utero‐placental system to support the foetus declines at advanced gestation,^4^ and this could potentially lead to increased pregnancy complications. Therefore, delivery at 39 or 40 weeks' gestation, when foetal lungs and other organ systems have matured, has been proposed to reduce adverse perinatal outcomes.^5^, ^6^

Several randomised trials and meta‐analyses have shown that labour induction between 39 and 41 weeks among uncomplicated singleton pregnancies results in lower rates of neonatal respiratory morbidity, meconium aspiration syndrome, low (<7) 5‐min Apgar score and perinatal death,^7^, ^8^, ^9^, ^10^, ^11^ while other studies report no difference or higher rates of adverse perinatal outcomes following induction compared with expectant management.^12^, ^13^

The time of delivery in low‐risk pregnancies is of particular concern as the clinician must balance the higher risks of respiratory morbidity and other neonatal complications associated with early‐term delivery with the risks of post‐maturity, including stillbirth, meconium aspiration and maternal complications that are higher at post‐term gestation.^14^ Increased rates of neonatal morbidity due to post‐maturity, which can influence risks of neurodevelopmental disorders, such as cerebral palsy and epilepsy are especially concerning.^15^ The most important knowledge gap in connection with obstetrics management for time of delivery in low‐risk pregnancies at ≥37 weeks' gestation relates to the absence of information on long‐term neurodevelopmental outcomes.^3^, ^16^ We, therefore, used data from the Swedish national population and health registries and examined associations between gestational age and risks of stillbirth, infant mortality, cerebral palsy (CP) and epilepsy in singleton births at 37–42 completed weeks among low‐risk pregnancies. We also assessed the potential mediating role of asphyxia‐related complications on the effect of gestational age at birth on infant mortality, CP and epilepsy.

## 2 METHODS

This study was based on singleton births in Sweden between 1998 and 2019 with data obtained from the Medical Birth Register.^17^ This database contains information on antenatal, obstetric and neonatal care that is prospectively recorded on standardised forms on more than 98% of all births in Sweden. Using the person‐unique national registration numbers of mothers and infants, data from the Birth Register were linked to several national registries. The nation‐wide National Patient Register^18^, ^19^ includes diagnostic codes and procedures on hospital inpatient care since 1987 and hospital outpatient care from 2001. The Cause of Death Register includes information on all deaths in Sweden.^20^ In the Medical Birth, Patient and Cause of Death Registers, diseases and deaths are, since 1997, coded using the Swedish version of the International Classification of Diseases, tenth revision (ICD‐10) 1997. Information on maternal education and country of origin was obtained from the Education Register and the Total Population Register, respectively.^21^, ^22^

### 2.1 Study population

Between 1 January 1998 and 31 December 2019, we identified 2,119,175 singleton live births and stillbirths at ≥37 completed gestational weeks from the Medical Birth Register. We excluded births with missing information on personal identification numbers (*n* = 2296), sex, date of birth and maternal age (*n* = 3). We further excluded infants born at ≥43+0 weeks (*n* = 9103), those in breech presentation and infants of mothers with chronic diseases and selected pregnancy complications^21^ (*n* = 334,504). All ICD‐10 codes used in the analyses are shown in Table S1. The final study population included 1,773,269 births.

### 2.2 Exposure

Gestational age (in completed weeks) was estimated using the following hierarchy: the date of early second trimester ultrasound (90.5%), the date of the last menstrual period (5.4%) and a postnatal assessment (4.1%). We evaluated outcomes at each gestational week by comparing delivery at a given gestational week with delivery at a later gestational week (i.e. expected management). The latter group thus included women who went on to have a spontaneous labour, an induction or a caesarean delivery at a later gestational age (a comparison more conducive to the clinical paradigm and better suited to guide clinical decision‐making). Thus, the effect of delivery at 37 weeks was assessed by comparing outcome rates among births at 37+0 to 37+6 weeks with those among births at 38+0 to 42+6 weeks and so forth.

### 2.3 Outcomes

We focussed on stillbirths at ≥37 completed gestational weeks (the Birth Register includes livebirths ≥22 weeks; stillbirths ≥28 weeks or later from 1998; and stillbirths ≥22 weeks from 2009 onwards). Infant mortality was defined as death within the first year after birth. Cases of CP and epilepsy were identified by the presence of one or more diagnostic codes for CP (ICD‐10 code G80) or epilepsy (ICD‐10 code G40) either in hospitalisation records or in outpatient visit records (diagnosis of epilepsy was restricted to the period after 27 days of age). Children with epilepsy who also had CP (*n* = 1169 infants) were not included in the epilepsy cohort. The date associated with the first record of epilepsy or CP was considered the date of diagnosis (up to 16 years of age).

### 2.4 Covariates

Maternal characteristics examined included age at delivery, country of birth, education, cohabitation with a partner, parity, height and smoking during pregnancy. Mothers who reported daily smoking at the first antenatal visit and/or at 30–32 gestational weeks were classified as smokers. Information on onset of labour (spontaneous, induced or caesarean delivery) was obtained from obstetric records, and the year of delivery was examined to address temporal changes in obstetric practice.

### 2.5 Statistical analysis

The distributions of maternal and infant characteristics were quantified for births occurring at each gestational week. We examined the association between gestational age and stillbirth, infant mortality, CP and epilepsy on both additive (risk difference) and multiplicative (risk ratio [RR]) scales, derived from fitting log‐linear Poisson regression models with robust variance (using an identity link for the risk difference and a log link for the risk ratio).^23^ In the multivariable analyses, estimates were adjusted for maternal factors (maternal age, height, country of origin, parity, education level and smoking), child's sex and year of birth. We also performed stratified analyses of feto‐infant mortality (i.e. stillbirth and/or infant mortality), CP and epilepsy by onset of labour (spontaneous, caesarean delivery and induced). In this analysis, rates of outcomes at any gestational week within each onset of labour category were compared with rates of outcome among all births at subsequent gestational weeks.

### 2.6 Causal mediation analysis

We considered asphyxia‐related neonatal conditions (e.g. convulsions, meconium aspiration and hypoxic ischaemic encephalopathy; Table S2 lists the ICD codes used), diagnosed at 0–27 days of age, as potential mediators for the effect of gestational age on infant mortality, CP and epilepsy (Figure S1, Tables S3 and S4). Therefore, we undertook causal mediation analyses based on a counterfactual framework^24^ to disentangle the association between gestational age and the outcomes (i.e. total effect) into the natural direct effect (the association between gestational age and the outcomes [infant mortality, CP and epilepsy] in the absence of asphyxia‐related conditions) and the natural indirect effect (the association operating through the mediators).^25^ We also estimated the controlled direct effect, which provided an estimate of the effect of gestational age on the outcomes that is not mediated through asphyxia‐related conditions. We additionally estimated the proportion of the total effect (on the RR scale) between gestational age and the outcome(s) that was mediated through asphyxia‐related conditions. All 95% CI estimates were based on 1,000 bootstrap samples. Log‐linear regression models and the mediation analysis were carried out in SAS (version 9.4; SAS Institute) using GENMOD and the CAUSALMED procedures, respectively.

### 2.7 Missing data

Since some of the covariates had missing values, we imputed missing data through multiple imputation using a chained equations approach.^26^ We assumed that the pattern of missing data was ‘missing at random’ and created 10 imputed data sets (after 200 burn‐in iterations). Results from 10 multiple imputation cycles were combined with the use of PROC MIANALYZE in SAS.

### 2.8 Sensitivity analysis

We performed several sensitivity analyses. First, since mediation methods were developed under a strict no‐unmeasured confounding assumption,^24^ we examined the robustness of causal effects to unmeasured confounders, by estimating an E‐value (defined as the maximal strength of association that an unmeasured confounder would need to have with the exposure and the outcome, to fully explain away an observed exposure–outcome association).^27^ Second, since information on body mass index (BMI) was missing in 149,013 (8.4%) women and information on major congenital malformations was only available among live births, adjustment for BMI, malformations (Table S2) and birthweight‐for‐gestational age was carried out in sensitivity analyses restricted to live births. BMI was calculated using weight measured at registration for antenatal care and self‐reported height. Birth weight‐for‐gestational age was estimated using the sex‐specific Swedish foetal growth reference.^28^

Third, women with hypertensive disorders of pregnancy were included in the expectant management group in sensitivity analyses, as gestational hypertension and preeclampsia can occur later in pregnancy and are a potential complication of expectant management. Fourth, as death prior to the diagnosis of CP or epilepsy could preclude a child from being diagnosed with either condition, we additionally quantified the adjusted association between gestational age at birth and a composite outcome of death or CP (i.e. stillbirth, infant death or CP) and a composite outcome of death or epilepsy (i.e. stillbirth, infant death or epilepsy). Fifth, to account for the varying lengths of follow‐up and since the probability of being diagnosed with CP and epilepsy changes with age, we calculated hazard ratios (HRs) and 95% CIs using Cox proportional hazard models. Stratified analyses by parity (0 or ≥1) were performed to investigate whether parity modified the association between gestational age and the composite outcomes.

### 2.9 Ethics approval

The study was approved by the Research Ethics Committee at the Karolinska Institutet, Stockholm, Sweden (No. 2020‐01545).

## 3 RESULTS

Mothers of infants born at 37–38 weeks were more likely to be younger (≤19 years), to smoke, to be shorter (≤159 cm), underweight (BMI < 18.5 kg/m^2^), multiparous (parity ≥4), to have a lower educational level and to have had a caesarean delivery, while infants born at 37–38 weeks of gestation were more often small‐for‐gestational age (<3rd percentile, Table 1).

**TABLE 1:** Maternal and birth characteristics according to gestational age, singleton term births in Sweden from 1998 to 2019

Maternal and birth characteristics	Gestational age (weeks)					
	Total	37–38	39	40	41	42
	No. (%)	No. (%)	No. (%)	No. (%)	No. (%)	No. (%)
Total	1,773,269	307,203 (17.3)	433,712 (24.5)	542,607 (30.6)	356,622 (20.1)	133,125 (7.5)
Maternal age (years)						
≤19	25,986 (1.5)	4925 (1.6)	6817 (1.6)	7794 (1.4)	4689 (1.3)	1761 (1.3)
20–24	225,587 (12.7)	38,699 (12.6)	57,383 (13.2)	70,151 (12.9)	43,558 (12.2)	15,796 (11.9)
25–29	556,933 (31.4)	92,668 (30.2)	136,880 (31.6)	174,290 (32.1)	111,777 (31.3)	41,318 (31.0)
30–34	614,224 (34.6)	103,544 (33.7)	148,901 (34.3)	188,528 (34.7)	125,719 (35.3)	47,532 (35.7)
≥35	350,539 (19.8)	67,367 (21.9)	83,731 (19.3)	101,844 (18.8)	70,879 (19.9)	26,718 (20.1)
Country of birth						
Nordic	1,374,136 (77.5)	231,604 (75.4)	329,491 (76.0)	424,733 (78.3)	283,611 (79.5)	10,4697 (78.6)
Non‐Nordic	397,395 (22.4)	75,316 (24.5)	103,787 (23.9)	117,319 (21.6)	72,675 (20.4)	28,298 (21.3)
Missing	1738 (0.1)	283 (0.1)	434 (0.1)	555 (0.1)	336 (0.1)	130 (0.1)
Education (years)						
≤9	150,369 (8.5)	30,301 (9.9)	37,881 (8.7)	43,568 (8.0)	27,515 (7.7)	11,104 (8.3)
10–11	206,415 (11.6)	39,651 (12.9)	51,261 (11.8)	60,978 (11.2)	39,400 (11.0)	15,125 (11.4)
12	447,716 (25.2)	78,359 (25.5)	110,132 (25.4)	137,010 (25.3)	89,329 (25.0)	32,886 (24.7)
13–14	256,973 (14.5)	44,061 (14.3)	62,206 (14.3)	78,964 (14.6)	52,226 (14.6)	19,516 (14.7)
≥15	694,337 (39.2)	111,818 (36.4)	167,925 (38.7)	216,859 (40.0)	144,734 (40.6)	53,001 (39.8)
Missing	17,459 (1.0)	3013 (1.0)	4307 (1.0)	5228 (1.0)	3418 (1.0)	1493 (1.1)
Year of delivery						
1998–02	361,569 (20.4)	63,674 (20.7)	86,065 (19.8)	110,973 (20.5)	72,798 (20.4)	28,059 (21.1)
2003–07	396,273 (22.3)	72,218 (23.5)	95,279 (22.0)	120,938 (22.3)	78,645 (22.1)	29,193 (21.9)
2008–12	420,789 (23.7)	73,083 (23.8)	104,505 (24.1)	129,897 (23.9)	83,268 (23.3)	30,036 (22.6)
2013–19	594,638 (33.5)	98,228 (32.0)	147,863 (34.1)	180,799 (33.3)	121,911 (34.2)	45,837 (34.4)
Smoking during pregnancy						
No	1,571,554 (88.6)	265,307 (86.4)	382,917 (88.3)	483,229 (89.1)	320,122 (89.8)	119,979 (90.1)
Yes	133,684 (7.5)	27,925 (9.1)	33,663 (7.8)	39,021 (7.2)	23,938 (6.7)	9137 (6.9)
Missing	68,031 (3.8)	13,971 (4.5)	17,132 (4.0)	20,357 (3.8)	12,562 (3.5)	4009 (3.0)
Mothers cohabits with partner						
Yes	1,584,819 (89.4)	271,589 (88.4)	387,584 (89.4)	486,996 (89.8)	319,768 (89.7)	118,882 (89.3)
No	101,244 (5.7)	18,514 (6.0)	24,137 (5.6)	29,343 (5.4)	20,405 (5.7)	8845 (6.6)
Missing	87,206 (4.9)	17,100 (5.6)	21,991 (5.1)	26,268 (4.8)	16,449 (4.6)	5398 (4.1)
Parity						
1	770,740 (43.5)	119,728 (39.0)	174,600 (40.3)	233,538 (43.0)	168,995 (47.4)	73,879 (55.5)
2	664,070 (37.4)	118,178 (38.5)	174,449 (40.2)	208,634 (38.5)	124,767 (35.0)	38,042 (28.6)
3	238,727 (13.5)	47,233 (15.4)	60,408 (13.9)	71,760 (13.2)	44,883 (12.6)	14,443 (10.8)
≥4	99,732 (5.6)	22,064 (7.2)	24,255 (5.6)	28,675 (5.3)	17,977 (5.0)	6761 (5.1)
Maternal height (cm)						
≤159	239,786 (13.5)	52,984 (17.2)	64,598 (14.9)	67,550 (12.4)	39,792 (11.2)	14,862 (11.2)
160–164	445,836 (25.1)	82,226 (26.8)	112,623 (26.0)	134,430 (24.8)	85,094 (23.9)	31,463 (23.6)
165–169	504,089 (28.4)	83,760 (27.3)	122,426 (28.2)	156,276 (28.8)	103,396 (29.0)	38,231 (28.7)
≥170	548,959 (31.0)	81,167 (26.4)	125,585 (29.0)	174,115 (32.1)	121,833 (34.2)	46,259 (34.7)
Missing	34,599 (2.0)	7066 (2.3)	8480 (2.0)	10,236 (1.9)	6507 (1.8)	2310 (1.7)
Maternal BMI (kg/m^2^)						
<18.5	41,278 (2.3)	8900 (2.9)	11,577 (2.7)	12,246 (2.3)	6573 (1.8)	1982 (1.5)
18.5–24.9	1,000,940 (56.4)	169,135 (55.1)	250,840 (57.8)	312,200 (57.5)	198,787 (55.7)	69,978 (52.6)
25–29.9	402,191 (22.7)	67,922 (22.1)	94,344 (21.8)	121,761 (22.4)	84,267 (23.6)	33,897 (25.5)
30–34.9	129,795 (7.3)	23,382 (7.6)	29,659 (6.8)	37,473 (6.9)	27,421 (7.7)	11,860 (8.9)
≥35	50,052 (2.8)	9460 (3.1)	10,781 (2.5)	14,063 (2.6)	10,640 (3.0)	5108 (3.8)
Missing	149,013 (8.4)	28,404 (9.2)	36,511 (8.4)	44,864 (8.3)	28,934 (8.1)	10,300 (7.7)
Infant sex						
Male	911,826 (51.4)	155,888 (50.7)	214,519 (49.5)	273,807 (50.5)	190,156 (53.3)	77,456 (58.2)
Female	861,443 (48.6)	151,315 (49.3)	219,193 (50.5)	268,800 (49.5)	166,466 (46.7)	55,669 (41.8)
Birth weight for gestational age (percentile)						
<3	19,970 (1.1)	4578 (1.5)	4215 (1.0)	5332 (1.0)	3982 (1.1)	1863 (1.4)
3 to <10	79,348 (4.5)	12,353 (4.0)	17,414 (4.0)	23,650 (4.4)	17,838 (5.0)	8093 (6.1)
10 to 90	1,461,941 (82.4)	242,470 (78.9)	357,058 (82.3)	452,566 (83.4)	298,352 (83.7)	111,495 (83.8)
>90 to 97	149,614 (8.4)	30,955 (10.1)	38,642 (8.9)	44,290 (8.2)	27,025 (7.6)	8702 (6.5)
>97	58,911 (3.3)	16,042 (5.2)	15,665 (3.6)	15,790 (2.9)	8737 (2.4)	2677 (2.0)
Missing	3485 (0.2)	805 (0.3)	718 (0.2)	979 (0.2)	688 (0.2)	295 (0.2)
Mode of delivery						
Vaginal non‐instrumental	1,431,191 (80.7)	223,558 (72.8)	359,036 (82.8)	467,640 (86.2)	288,156 (80.8)	92,801 (69.7)
Vaginal instrumental	128,000 (7.2)	13,819 (4.5)	24,374 (5.6)	39,783 (7.3)	33,988 (9.5)	16,036 (12.0)
Planned caesarean section	92,639 (5.2)	52,106 (17.0)	31,453 (7.3)	4391 (0.8)	2837 (0.8)	1852 (1.4)
Emergency caesarean section	111,772 (6.3)	14,904 (4.9)	16,628 (3.8)	28,408 (5.2)	30,090 (8.4)	21,742 (16.3)
Missing	9667 (0.5)	2816 (0.9)	2221 (0.5)	2385 (0.4)	1551 (0.4)	694 (0.5)

There were 2736 cases of stillbirth, 1981 cases of infant death, 2649 cases of CP and 12,877 cases of epilepsy. The median age at diagnosis of CP was 2.1 years (interquartile range [IQR] 1.1, 4.4 years) and 5.4 years (IQR 2.2, 9.2 years) for epilepsy.

**FIGURE 1:**
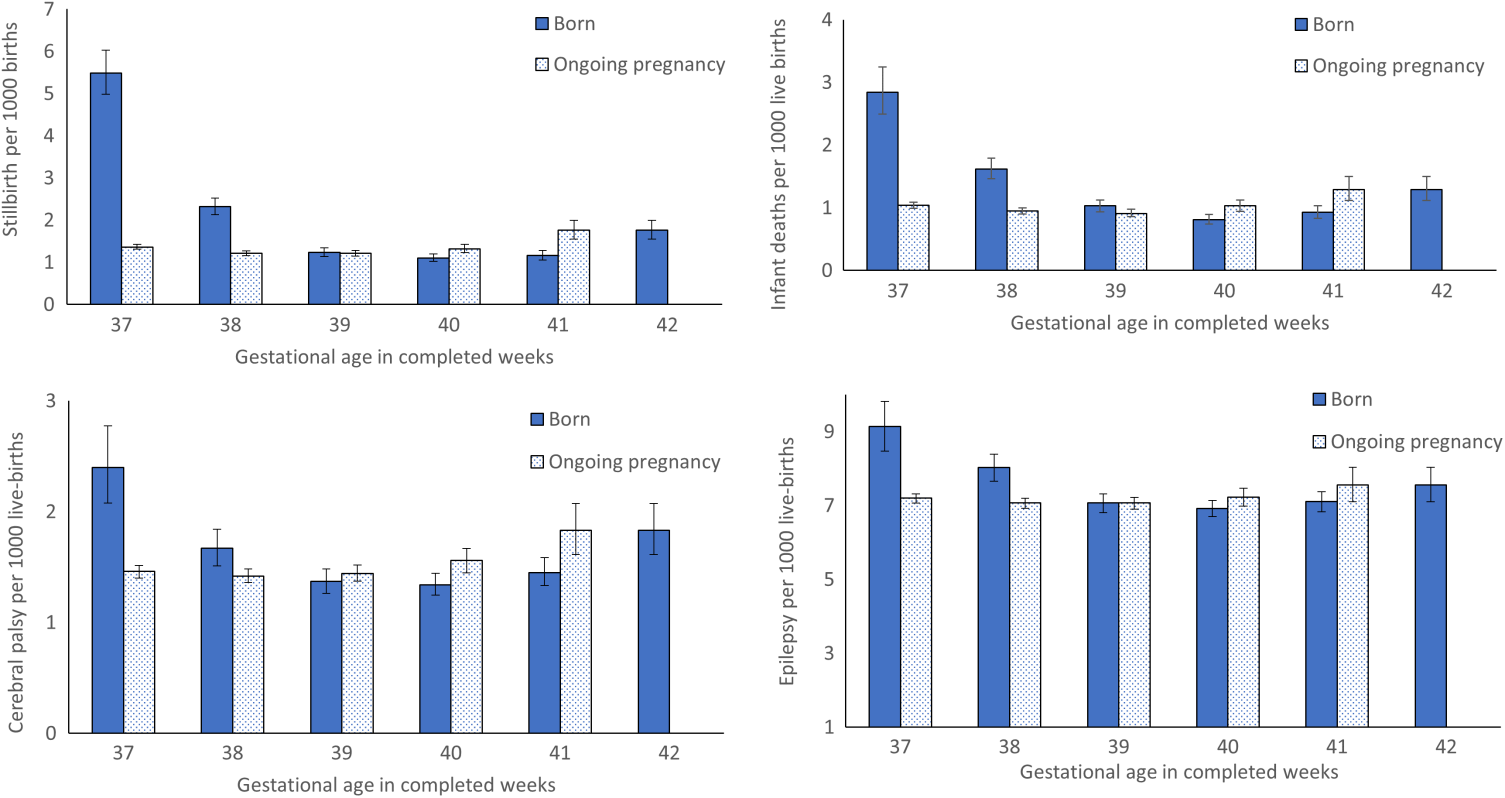
Gestational age and rates of stillbirth, infant mortality, cerebral palsy and epilepsy, Sweden, 1998–2019

Among births at a given gestational week, stillbirth rates decreased from 37 to 40 weeks before increasing at 41 and 42 weeks' gestation (Figure 1). Compared with births at a later gestational week, risks of stillbirth were higher among births at 37 weeks of gestation and 38 weeks of gestation. The adjusted risk ratio for stillbirth among births at 39 weeks' gestation was similar to the risk of stillbirth among births at ≥40 weeks (Table 2). The absolute rate differences for stillbirth were lower among births that occurred at 40 weeks and at 41 weeks compared with those that occurred at >40 and >41 week's gestation, respectively.

**TABLE 2:** Gestational age at birth and relative risks of stillbirth, infant mortality, cerebral palsy and epilepsy among singleton term births in Sweden, 1998,2019

	Deliveries	No. of cases	Unadjusted relative risk (95% CI)	Adjusted relative risk (95% CI)^a^	Adjusted rate difference (95% CI)^a^
Stillbirth					
37 weeks vs ≥38 week	77,038	422	4.01 (3.62, 4.45)	3.88 (3.47, 4.34)	3.85 (3.32, 4.38)
38 weeks vs ≥39 week	230,165	533	1.90 (1.73, 2.10)	1.96 (1.76, 2.17)	1.10 (0.89, 1.31)
39 weeks vs ≥40 week	433,712	535	1.02 (0.92, 1.13)	1.01 (0.91, 1.13)	0.01 (−0.11, 0.14)
40 weeks vs ≥41 week	542,607	598	0.83 (0.75, 0.93)	0.85 (0.75, 0.96)	−0.18 (−0.32, −0.05)
41 weeks vs 42 week	356,622	414	0.66 (0.56, 0.77)	0.68 (0.58, 0.81)	−0.51 (−0.77, −0.26)
Infant mortality					
37 weeks vs ≥38 week	77,038	219	2.72 (2.35, 3.14)	2.45 (2.10, 2.86)	1.57 (1.18, 1.96)
38 weeks vs ≥39 week	230,165	373	1.69 (1.50, 1.90)	1.61 (1.42, 1.82)	0.58 (0.41, 0.76)
39 weeks vs ≥40 week	433,712	445	1.12 (1.00, 1.25)	1.04 (0.92, 1.17)	0.04 (to −0.08, 0.15)
40 weeks vs ≥41 week	542,607	441	0.79 (0.70, 0.90)	0.79 (0.69, 0.90)	−0.21 (−0.34, −0.09)
41 weeks vs 42 week	356,622	331	0.72 (0.60, 0.86)	0.75 (0.62, 0.91)	−0.31 (−0.54, −0.09)
Cerebral palsy^b^					
37 weeks vs ≥38 week	76,616	184	1.64 (1.41, 1.91)	1.60 (1.37, 1.87)	0.92 (0.55, 1.29)
38 weeks vs ≥39 week	229,632	383	1.16 (1.04, 1.30)	1.19 (1.06, 1.33)	0.27 (0.08, 0.46)
39 weeks vs ≥40 week	433,177	593	0.95 (0.86, 1.04)	0.95 (0.86, 1.04)	−0.08 (−0.21, 0.06)
40 weeks vs ≥41 week	542,009	728	0.86 (0.78, 0.96)	0.88 (0.79, 0.98)	−0.18 (−0.33, −0.03)
41 weeks vs 42 week	356,208	518	0.80 (0.68, 0.93)	0.80 (0.69, 0.94)	−0.36 (−0.63, −0.08)
Epilepsy^b^					
37 weeks vs ≥38 week	76,616	699	1.27 (1.17, 1.37)	1.24 (1.15, 1.34)	1.80 (1.08, 2.53)
38 weeks vs ≥39 week	229,632	1841	1.13 (1.08, 1.19)	1.12 (1.07, 1.18)	0.90 (0.49, 1.31)
39 weeks vs ≥40 week	433,177	3057	1.00 (0.96, 1.04)	1.01 (0.97, 1.06)	0.07 (−0.24, 0.38)
40 weeks vs ≥41 week	542,009	3748	0.96 (0.91, 1.00)	0.96 (0.91, 1.00)	−0.31 (−0.65, 0.03)
41 weeks vs 42 week	356,208	2528	0.94 (0.87, 1.01)	0.94 (0.87, 1.01)	−0.48 (−1.05, 0.08)

Note

Absolute rate differences represent the excess number of neonatal deaths per 1000 births.

^a^ Adjusted for maternal age, country of origin, education level, cohabitation with a partner, parity, height, smoking during pregnancy, child's sex and year of delivery.

^b^ Live birth only.

Among live births at a given gestational week, infant mortality rates decreased from 37 to 40 weeks, before increasing at 41 and 42 weeks' gestation (Figure 1). Adjusted risks and the absolute risk differences in infant mortality were higher among births at 37 and 38 weeks of gestation compared with live births at a later gestational week (Table 2). The adjusted risks for infant mortality were 20% and 25% lower among live births that occurred at 40 and 41 weeks, compared with those that occurred at >40 and >41 week's gestation, respectively.

The rate of CP was lowest among live births at 40 weeks' gestation, with the highest rates at 37 weeks and at 42 weeks (Figure 1). Infants born at 37 weeks and 38 weeks had higher adjusted rate ratios and absolute risk differences for CP than infants born at ≥38 weeks and ≥39, respectively. The risk for CP was not elevated among infants born at 39 weeks, compared with infants born at ≥40 weeks. Infants born at 40 weeks and infants born at 41 weeks had lower risks for CP compared with those born later (Table 2).

The rate of epilepsy among live births declined from 37 to 40 weeks of gestation, before showing a slight increase at 42 weeks (Figure 1). Infants born at 37 weeks and 38 weeks had higher adjusted rate ratios of epilepsy compared with those born at ≥38 and ≥39 weeks, respectively (Table 2). Live births at 39, 40 and 41 weeks gestation did not have higher risks of epilepsy compared with infants born at later gestational ages.

Among those with spontaneous onset of labour, 5% had an emergency caesarean delivery, 8% had an instrumental vaginal delivery, and 87% had non‐instrumental vaginal delivery. Births that occurred after spontaneous onset of labour at 37 weeks' gestation had elevated risks of feto‐infant mortality, CP and epilepsy compared with births at subsequent gestational weeks (Table 3). However, spontaneous births at 39, 40 and 41 weeks had lower risks of feto‐infant mortality and CP compared with those born at a later gestation. Infants born following a caesarean delivery had substantially higher risks of feto‐infant mortality, CP and epilepsy, with the highest adjusted RRs at 37 and 40 weeks compared with births that occurred at later gestational ages. Among the induced group, 17% had an emergency caesarean delivery, 10% had an instrumental vaginal delivery, and 73% had vaginal non‐instrumental delivery. Births following induction of labour at any gestational week had higher risks of feto‐infant mortality compared with births at subsequent gestational weeks, and higher risks of CP and epilepsy among live births at 37, 38, and 39 week's gestation compared with live births at subsequent gestation (Table 3).

**TABLE 3:** Gestational age at birth and relative risks (RRs) and 95% confidence intervals (CIs) of feto‐infant mortality, cerebral palsy and epilepsy stratified by onset of delivery among singleton term births in Sweden, 1998–2019

Onset of labour: gestational week	Feto‐infant mortality		Cerebral Palsy		Epilepsy	
	No. (per 1000 births)	Adjusted Relative Risk^a^	No. (per 1000 births)	Adjusted Relative Risk^a^	No. (per 1000 births)	Adjusted Relative Risk^a^
Spontaneous labour						
37 weeks vs ≥38 week	240 (4.1)	1.65 (1.43, 1.90)	123 (2.1)	1.41 (1.17, 1.70)	473 (8.2)	1.11 (1.00, 1.22)
38 weeks vs ≥39 week	405 (2.6)	1.18 (1.06, 1.32)	239 (1.5)	1.08 (0.94, 1.24)	1221 (7.7)	1.07 (1.01, 1.14)
39 weeks vs ≥40 week	550 (1.5)	0.67 (0.61, 0.75)	483 (1.3)	0.91 (0.81, 1.01)	2606 (7.0)	0.99 (0.94, 1.04)
40 weeks vs ≥41 week	615 (1.2)	0.54 (0.49, 0.60)	634 (1.3)	0.84 (0.75, 0.94)	3425 (6.9)	0.95 (0.90, 0.99)
41 weeks vs 42 week	476 (1.5)	0.53 (0.46, 0.61)	438 (1.4)	0.77 (0.65, 0.90)	2227 (7.0)	0.92 (0.85, 0.99)
Caesarean delivery						
37 weeks vs ≥38 week	73 (10.7)	4.10 (3.19, 5.26)	29 (4.3)	2.94 (2.01, 4.31)	76 (11.2)	1.47 (1.16, 1.86)
38 weeks vs ≥39 week	116 (2.6)	1.21 (0.99, 1.48)	92 (2.0)	1.48 (1.19, 1.85)	385 (8.5)	1.19 (1.07, 1.33)
39 weeks vs ≥40 week	69 (2.2)	1.08 (0.84, 1.40)	46 (1.5)	1.09 (0.78, 1.52)	191 (6.1)	1.08 (0.93, 1.25)
40 weeks vs ≥41 week	46 (10.5)	4.99 (3.69, 6.75)	27 (6.2)	3.73 (2.45, 5.69)	35 (8.0)	1.23 (0.88, 1.72)
41 weeks vs 42 week	15 (5.3)	2.13 (1.26, 3.60)	12 (4.2)	2.60 (1.44, 4.70)	26 (9.2)	1.19 (0.79, 1.80)
Induced						
37 weeks vs ≥38 week	317 (27.3)	11.0 (9.70, 12.4)	30 (2.6)	1.88 (1.29, 2.76)	142 (12.5)	1.85 (1.55, 2.19)
38 weeks vs ≥39 week	369 (15.3)	7.22 (6.42, 8.13)	48 (2.0)	1.46 (1.08, 1.98)	212 (8.9)	1.37 (1.19, 1.58)
39 weeks vs ≥40 week	334 (11.4)	5.15 (4.54, 5.85)	55 (1.9)	1.43 (1.08, 1.88)	236 (8.2)	1.23 (1.07, 1.41)
40 weeks vs ≥41 week	361 (10.1)	4.16 (3.65, 4.73)	58 (1.6)	1.18 (0.90, 1.54)	269 (7.6)	1.09 (0.96, 1.25)
41 weeks vs 42 week	240 (6.8)	2.29 (1.92, 2.73)	60 (1.7)	0.98 (0.73, 1.32)	255 (7.2)	1.03 (0.89, 1.19)

^a^ Adjusted for maternal age, country of origin, education level, cohabitation with a partner, parity, height, smoking during pregnancy, child's sex and year of delivery.

### 3.1 Causal mediation analyses

We examined the impact of asphyxia‐related conditions on the association between gestational age and infant mortality, CP and epilepsy (Table 4). The RRs for the natural direct and natural indirect (mediated) effects of live birth at 40 weeks of gestation on infant mortality were 0.88 (95% CI 0.69, 0.99) and 0.93 (95% CI 0.91, 0.95), respectively. Asphyxia‐related neonatal conditions mediated 35% (95% CI 3, 46) of the effect of live birth at 41 weeks' gestation (vs 42 weeks) on CP and did not appear to mediate the association between other categories of gestational age and CP. For epilepsy, the proportion mediated could not be estimated, since the RRs for natural direct effect and the natural indirect effects are on opposite direction (Table 4).

**TABLE 4:** Causal mediation analyses to estimate the impact of asphyxia‐related neonatal conditions^a^ on the association between gestational age and infant mortality, cerebral palsy and epilepsy, liveborn singleton term infants in Sweden, 1998–2019

Gestational age	Risk ratio (95% CI)				
	Total effect	Controlled direct effect	Natural direct effect	Natural indirect effect	Percentage mediated (%) (95% CI)
Infant Mortality					
37 weeks vs ≥38 week	2.45 (2.02, 3.03)	2.62 (2.18, 3.06)	2.50 (2.11, 2.89)	0.98 (0.97, 0.99)	__^b^
38 weeks vs ≥39 week	1.62 (1.31, 1.82)	1.71 (1.36, 1.94)	1.71 (1.37, 1.91)	0.95 (0.93, 0.97)	__^b^
40 weeks vs ≥41 week	0.82 (0.64, 0.92)	0.88 (0.69, 1.02)	0.88 (0.69, 0.99)	0.93 (0.91, 0.95)	35 (4, 50)
41 weeks vs 42 week	0.73 (0.59, 1.00)	0.74 (0.57, 1.04)	0.79 (0.63, 1.07)	0.93 (0.91, 0.97)	20 (−8, 60)
Cerebral palsy					
37 weeks vs ≥38 week	1.63 (1.36, 2.03)	1.70 (1.41, 2.19)	1.69 (1.40, 2.08)	0.96 (0.95, 1.00)	__^b^
38 weeks vs ≥39 week	1.22 (1.00, 1.34)	1.28 (1.01, 1.41)	1.32 (1.08, 1.46)	0.92 (0.89, 0.95)	__^b^
41 weeks vs 42 week	0.79 (0.60, 0.91)	0.81 (0.58, 0.96)	0.87 (0.65, 0.98)	0.92 (0.89, 0.96)	35 (3, 46)
Epilepsy					
37 weeks vs ≥38 week	1.24 (1.06, 1.32)	1.24 (1.07, 1.32)	1.24 (1.07, 1.32)	1.00 (0.99, 1.00)	__^b^
38 weeks vs ≥39 week	1.13 (1.03, 1.18)	1.14 (1.03, 1.18)	1.14 (1.03, 1.18)	0.99 (0.99, 1.00)	__^b^

Note

RRs are adjusted for maternal age, country of origin, education level, cohabitation with a partner, parity, height, smoking during pregnancy, child's sex and year of delivery. All 95% CI estimates are based on 1000 bootstrap resamples. The exposure–mediator interaction term was retained in the mediation analysis. CI, confidence intervals; RR, risk ratio.

^a^ Asphyxia‐related neonatal conditions included convulsions, meconium aspiration and hypoxic ischemic encephalopathy.

^b^ Estimates are not shown because the RRs for the natural direct effect and the natural indirect effect are on opposite directions.

### 3.2 Sensitivity analyses

Our primary results were unchanged using multiple imputations of missing data (Table S5). Adjustment for maternal BMI, major congenital malformations and birthweight‐for‐gestational age among live births did not substantially influence the results (Table S6). Including women diagnosed with pregnancy‐induced hypertensive disorders in pregnancy in the expectant management group further strengthened the associations between gestational age at birth and the outcomes (Table S7). Analyses of associations between gestational age at birth and composite death or CP (Table S8) and composite death or epilepsy (Table S9) showed similar effects with enhanced precision of the estimates. In the sensitivity analyses, the E‐value for the RR and the lower 95% CI were greater in magnitude compared with the observed estimates. These results suggest that the casual mediation parameters were robust to unmeasured confounding (Table S10). Hazard ratios from Cox regression showed similar results as the primary analyses, indicating that the effects of loss to follow‐up, that is, death or emigration, were minimal (Table S11). Lastly, stratified analyses by parity showed similar findings among primiparae and multiparae women. However, the risk of CP was not increased at any gestation among live births to primiparous women (Table S12).

## 4 COMMENT

### 4.1 Principal findings

In this nationwide cohort study of low‐risk pregnancies at term gestation, we found that risks of stillbirth, infant mortality, CP and epilepsy were increased among births at 37 and 38 weeks' gestation, compared with births at ≥38 weeks and ≥39 weeks, respectively. There were no differences in risks among births at 39 weeks' gestation, compared with births at ≥40 weeks. Risks of stillbirth, infant mortality and CP were reduced among births at 40 and 41 weeks' gestation, compared with births at ≥41 weeks and 42 weeks, respectively. Associations between late‐term delivery and infant mortality and CP were partly mediated by asphyxia‐related neonatal complications. Taken together, these findings suggest that delivery between 39+0 and 40+6 week's gestation represents the optimal time of delivery for low‐risk pregnancies.

### 4.2 Strengths of the study

This study has several strengths. The population‐based study design, together with the prospectively and independently collected information on exposure and outcomes and other high‐quality registry data, minimised the possibility of selection and information bias. Our analyses compared the rates of outcomes of births at each gestational week with the rates of outcomes among those born at subsequent gestational weeks. This allowed us to measure the effect of early‐term delivery versus expectant management at different gestational weeks and to directly address the question most relevant for clinical decision‐making and pregnancy management among low‐risk women. In addition, we quantified the effects on composite outcomes that included death or CP, and death or epilepsy (thereby addressing the issues related to outcome‐dependent censoring and competing risks).^29^

### 4.3 Limitation of the data

Some study limitations also deserve consideration. Despite limiting the study to low‐risk pregnancies, it is likely that a higher proportion of women who were induced or had caesarean delivery at early vs. late term had some unreported or unrecognised pregnancy complications. Such misclassification would have resulted in a higher proportion of compromised births at 37 and 38 weeks and an overestimation of the relative risk for stillbirth, infant death, CP and epilepsy at 37 and 38 weeks' gestation. Our study focussed on two important neurodevelopmental disorders, namely CP and epilepsy, and further studies are needed to assess effects related to intellectual disability and other disorders. Also, during the study period, some obstetric practices would have changed, including pregnancy dating, although we included year of delivery in our models to account for these potential secular changes in maternity care practices. Finally, using early second trimester ultrasonography, instead of a first trimester ultrasonography to estimate gestational age, could have resulted in less precision.^30^

### 4.4 Interpretation

Consistent with our findings, increased risks of neonatal mortality, intellectual deficits, poorer school performance and long‐term neurological disorders have been found in children born at early term (37–38 weeks).^31^, ^32^, ^33^, ^34^ Stillbirth risk increases with gestational age in term and especially post‐term pregnancies, and foetal growth restriction likely explains a part of this increased risk.^35^ Among births at a specific gestational week, an approximate U‐shaped pattern is evident in the gestational age‐specific risks of stillbirth, infant mortality, CP and epilepsy, with the highest risks at 37 weeks and 42 weeks.^34^, ^36^ These patterns arise because infants born at early term may be vulnerable due to physiological immaturity, while post‐term infants may be compromised due to post‐maturity. While some adverse outcomes among early‐term births may be attributed to immaturity, a large proportion are likely a consequence of pregnancy complications or underlying conditions that led to early delivery. A related issue arises because low‐risk women in non‐experimental studies can have differential distributions of pregnancy complications by place of birth, gestational age and other factors.^37^ This makes non‐experimental studies less suitable than randomised trials^9^ for identifying an optimal gestational age for delivery. Still, feasibility issues may limit the number of options explored with experimental designs.

The findings of this study add to the growing evidence that infants born between 39 and 40 weeks have the best perinatal and long‐term neurodevelopmental outcomes.^7^, ^8^, ^11^, ^38^, ^39^ In our study, the rates of stillbirth, infant mortality and CP were higher among infants born at 41 and 42 weeks' gestation than in infants born at 40 weeks, whereas the rate of epilepsy was not. Asphyxia‐related conditions explained part of the increased risk of infant mortality and CP at late‐term (41–42 weeks) gestation. Morbidity due to late gestation diseases, such as hypoxic ischaemic encephalopathy, meconium aspiration and neonatal seizures, showed a pronounced increase from term gestation onwards and contributed to the pathogenesis of infant mortality and neurological complications.^34^, ^40^, ^41^ Animal and human studies show that the ability of the utero‐placental system to support the foetus declines with advancing gestation^4^: although blood flow to the uterus increases as pregnancy advances, normalised blood flow (i.e. blood flow per unit foetal weight) falls.^42^, ^43^ These changes likely underlie the increase in adverse perinatal outcomes, including intrauterine growth restriction, stillbirth, neonatal death and neurological morbidity at late‐term and post‐term gestation, leaving 39 and 40 weeks' gestation as the optimal time for delivery.

## 5 CONCLUSIONS

In summary, our findings provide evidence to support the proposition that the time of delivery affects foetal and infant death and long‐term morbidity: delivery of low‐risk pregnancies between 37 and 38 weeks increases the risks of death or neurological complications, while delivery at 39 and 40 weeks pregnancies results in the lowest risk of adverse foetal, infant and neurodevelopmental outcomes. It is important that clinical practice accounts for both short‐ and long‐term risks and benefits of continuing pregnancy beyond 40 weeks' gestation.

## AUTHOR CONTRIBUTION

NR had full access to all of the data in the study and takes full responsibility for the integrity of the data and the accuracy of the data analysis (guarantors of this work). NR, GM and KSJ conceived and designed the study. All authors interpreted the data and critically revised the manuscript for important intellectual content. NR drafted the manuscript and carried out the statistical analysis. NR obtained funding.

## CONFLICT OF INTEREST

The authors report no conflicts of interest.

## Supporting information

Supplementary Material

## Data Availability

Data available on request due to privacy/ethical restrictions.
