# Supplementary material for: Time of delivery among low‐risk women at 37–42 weeks of gestation and risks of stillbirth and infant mortality, and long‐term neurological morbidity
Source: Paediatr Perinat Epidemiol. 2022 Mar 4;36(4):577–87. doi: 10.1111/ppe.12868 (PMC9314589; doi:10.1111/ppe.12868)
Supplement: Supplementary file 1 — Supplementary Material [file PPE-36-577-s001.docx]

**SUPPLEMENTARY Web appendix**

| **Table of Contents** | **Page** |
| --- | --- |
| **Table S1.** ICD-10 codes for maternal morbidity | **2** |
| **Table S2.** ICD-10 codes for neonatal morbidity. | **3** |
| **Figure S1.** Simplified directed acyclic graph depicting the relation between gestational age and infant mortality, CP and epilepsy with asphyxia related conditions as the mediator and unmeasured confounders. | **4** |
| **Table S3.** Gestational age at birth and risk ratios (RRs) and 95% confidence intervals (CIs) for asphyxia-related neonatal complications in liveborn singleton term infants born in Sweden, 1998-2019 | **5** |
| **Table S4.** Asphyxia-related neonatal complications and risk ratios (RRs) and 95% confidence intervals (CIs) for infant mortality, cerebral palsy and epilepsy in liveborn singleton term infants born in Sweden, 1998-2019 | **6** |
| **Table S5.** Multiple imputation analysis for gestational age at birth and risk ratios for infant mortality, cerebral palsy and epilepsy in term singleton infants born in Sweden, 1998-2019 | **7** |
| **Table S6.** Gestational age at birth and risk ratios (RRs) and 95% confidence intervals (CIs) for infant mortality, cerebral palsy and epilepsy, adjusting for maternal body mass index, birth weight for gestational age, and congenital malformations in liveborn singleton term infants born in Sweden, 1998-2019 | **8** |
| **Table S7.** Gestational age at birth and risk ratios (RRs) and 95% confidence intervals (CIs) for infant mortality, cerebral palsy and epilepsy, in sensitivity analyses including women with hypertensive diseases during pregnancy, singleton term infants born in Sweden, 1998-2019 | **7** |
| **Table S8.** Gestational age at birth and rates of cerebral palsy (composite outcome) in term singleton infants born in Sweden, 1998-2019 | **10** |
| **Table S9.** Gestational age at birth and rates of epilepsy (composite outcome) in term singleton infants born in Sweden, 1998-2019 | **11** |
| **Table S10.** Robustness to unmeasured confounding (E-values) of the total adjusted risk ratio expressing the relation between gestational age and infant mortality, cerebral palsy, and epilepsy, liveborn singleton term infants in Sweden, 1998–2019 | **12** |
| **Table S11.** Gestational age at birth and hazard ratios for cerebral palsy and epilepsy in liveborn singleton term infants born in Sweden, 1998-2019 | **13** |
| **Table S12.** Gestational age at birth and relative risks (RRs) and 95% confidence intervals (CIs) for stillbirth, infant mortality, cerebral palsy and epilepsy stratified by parity (0 or ≥1) among singleton term births in Sweden, 1998-2019 | **14** |

| **Table S1.** ICD-10 Codes for deriving the Obstetric Comorbidity Index | |
| --- | --- |
| **Co-morbidity** | **ICD10-CA Code** |
| Alcohol abuse | F10 |
| Asthma | J44, J45 |
| Cardiac Valvular Disease | I05-I09, I34-I39 |
| Chronic Congestive Heart Failure | I50.0 |
| Chronic Ischemic Heart Disease | I20, I25 |
| Chronic Renal Disease | N02.2, N03-N05, N08, N17.1, |
|  | N17.2, N18, N25 |
| Congenital Heart Disease | Q20-Q26, O99.4 |
| Drug Abuse | F11-F16, F18, F19 |
| Gestational Hypertension | O13, O16 |
| Human Immunodeficiency Virus | B20, B24, O98.7, Z21 |
| Mild/Unspecified Pre-Eclampsia | O11, O14 |
| Multiple Gestation | O30, O31, Z37.2-Z37.7, Z37.90 |
| Placenta Previa | O44 |
| Pre-Existing Diabetes Mellitus | E10, E11, O24.5-O24.7 |
| Pre-Existing Hypertension | I10-I15, O10 and O11+checkbox* |
| Previous Cesarean Delivery | O34.20 +checkbox** |
| Pulmonary Hypertension | I27.0, I27.2, I27.8, I27.9 |
| Pre-Eclampsia | O14, O15 |
| Sickle Cell Disease | D56, D57 |
| Systemic Lupus Erythematosus | M32 |
| ^*^Essential hypertension is also recorded in a checkbox in the prenatal record at first prenatal visit. | |
| ^**^Previous cesarean delivery is also recorded on admission to delivery. | |

**Table S2.** ICD-10 codes for neonatal morbidity

| **Diseases** | **ICD-10 codes** |
| --- | --- |
| **Major malformation^†^** | Q00-Q99, excluding: Q170, Q175, Q180, Q181, Q250, Q270, Q314, Q320, Q381, Q523, Q530-Q539, Q650-Q659, Q665-Q669, Q690, Q699, Q703, Q760, Q799, Q825, Q829 |
| **Neonatal morbidity**^‡^ |  |
| Asphyxia-related morbidity: |  |
| Meconium aspiration | P24.0 |
| Convulsions | P90 |
| Hypoxic ischemic encephalopathy  and related conditions | P910, P913-P916, P918, P919 |

Diseases were defined using the Swedish version of the International Classification of Diseases, ninth and tenth revision (ICD-10). Sweden used the tenth revision (ICD-10) has been used since 1997.

^†^Diagnosis of malformations includes diagnostic codes recorded in the Medical Birth Register, the Patient Register (also including out-patient hospital care from 2001) or the Cause of Death Register before 1 year of age (0-364 days). Minor (excluded) malformations are defined by the Swedish National Board of Health and Welfare (see <https://www.socialstyrelsen.se/globalassets/sharepoint-dokument/dokument-webb/ovrigt/diagnoser-som-inte-ska-rapporteras-om-fosterskador.pdf>)

^‡^Diagnoses of neonatal morbidity are from Medical Birth Register or the Patient Register (in-patient hospital care, admission date at 0-27 days of age).

**Figure S1.** Simplified directed acyclic graph depicting the relation between gestational age and infant mortality, CP and epilepsy with asphyxia-related conditions as the mediator and unmeasured confounders.


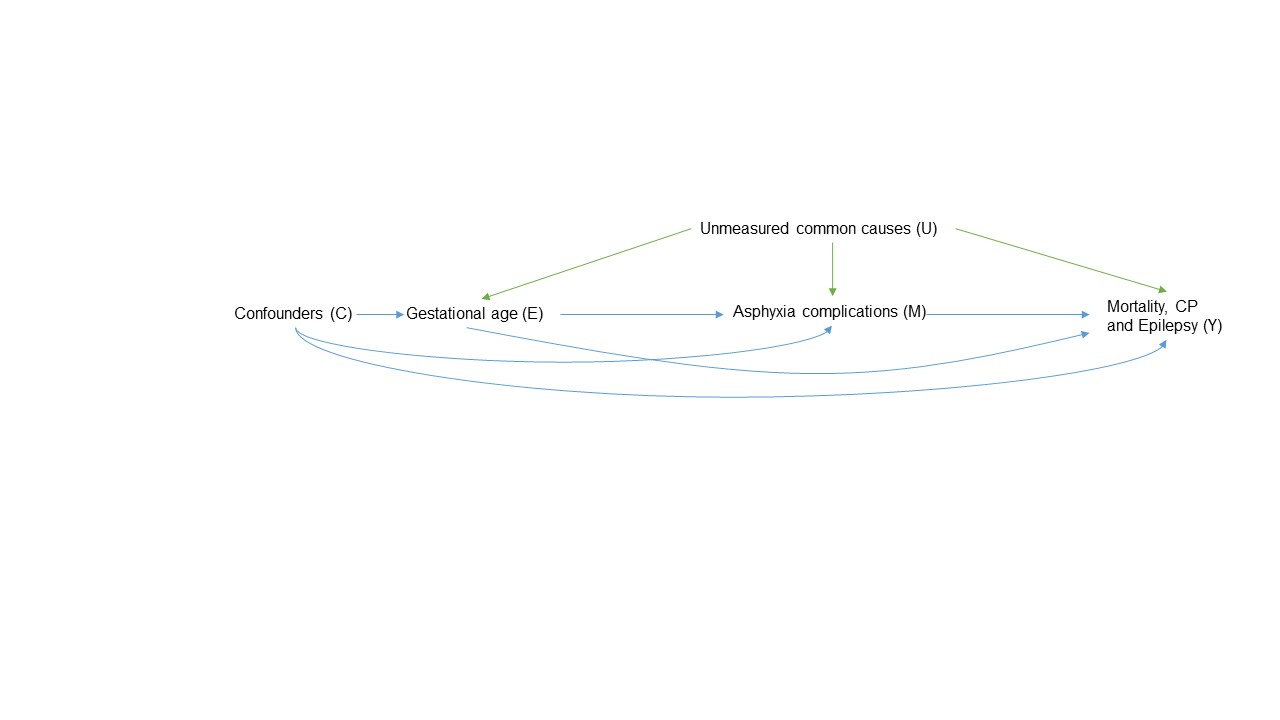


| **Table S3.** Gestational age at birth and risk ratios (RRs) and 95% confidence intervals (CIs) for asphyxia-related neonatal complications in liveborn singleton term infants born in Sweden, 1998-2019 | |
| --- | --- |
| **Comparison group** | **Asphyxia related neonatal complications*** |
|  | **Risk Ratio (95%CI)** ^†^ |
| 37 weeks vs ≥38 weeks | 0.78 (0.68,0.90) |
| 38 weeks vs ≥39 weeks | 0.63 (0.57,0.69) |
| 39 weeks vs ≥40 weeks | 0.56 (0.52,0.60) |
| 40 weeks vs ≥41 weeks | 0.59 (0.55,0.63) |
| 41 weeks vs 42 weeks | 0.69 (0.64,0.75) |

^†^ Adjusted for maternal age, country of origin, education level, cohabitation with a partner, parity, height, smoking during pregnancy, child’s sex, and year of delivery.

*Asphyxia related neonatal conditions included convulsions, meconium aspiration, and hypoxic ischemic encephalopathy

| **Table S4.** Gestational age at birth and risk ratios (RRs) and 95% confidence intervals (CIs) for infant mortality, cerebral palsy and epilepsy, adjusting for maternal body mass index, birth weight for gestational age and malformation in liveborn singleton term infants born in Sweden during 1998-2019 | | | |
| --- | --- | --- | --- |
| **Comparison group** | **Infant Mortality** | **Cerebral Palsy** | **Epilepsy** |
|  | **Risk Ratio (95%CI)** ^†^ | **Risk Ratio (95%CI)** ^†^ | **Risk Ratio (95%CI)** ^†^ |
| Asphyxia related complications | 52.66 (46.12,60.13) | 79.47 (72.24,87.42) | 7.70 (6.87,8.64) |

^†^ Adjusted for maternal age, country of origin, education level, cohabitation with a partner, parity, height, smoking during pregnancy, child’s sex, and year of delivery.

| **Table S5.** Multiple imputation analysis for gestational age at birth and risk ratios for stillbirth, infant mortality, cerebral palsy and epilepsy in term singleton infants born in Sweden, 1998-2019 | | | | |
| --- | --- | --- | --- | --- |
| **Comparison group** | **Stillbirth** | **Infant Mortality** | **Cerebral Palsy** | **Epilepsy** |
|  | **Risk Ratio (95%CI)** ^†^ | **Risk Ratio (95%CI)** ^†^ | **Risk Ratio (95%CI)** ^†^ | **Risk Ratio (95%CI)** ^†^ |
| 37 weeks vs ≥38 weeks | 3.84 (3.46, 4.27) | 2.52 (2.18, 2.90) | 1.56 (1.34, 1.82) | 1.22 (1.13, 1.32) |
| 38 weeks vs ≥39 weeks | 1.88 (1.70, 2.07) | 1.62 (1.45, 1.82) | 1.17 (1.04, 1.30) | 1.11 (1.06, 1.17) |
| 39 weeks vs ≥40 weeks | 1.03 (0.93, 1.14) | 1.10 (0.98, 1.23) | 0.96 (0.87, 1.05) | 1.01 (0.97, 1.05) |
| 40 weeks vs ≥41 weeks | 0.86 (0.77, 0.96) | 0.79 (0.69, 0.90) | 0.89 (0.80, 0.98) | 0.96 (0.91, 1.00) |
| 41 weeks vs 42 weeks | 0.69 (0.58, 0.81) | 0.73 (0.61, 0.88) | 0.82 (0.70, 0.96) | 0.95 (0.88, 1.02) |

^†^Adjusted for attained age of child, maternal age, country of origin, education level, cohabitation with a partner, parity, height, smoking during pregnancy, child’s sex and year of delivery.

| **Table S6.** Gestational age at birth and risk ratios (RRs) and 95% confidence intervals (CIs) for infant mortality, cerebral palsy and epilepsy, adjusting for maternal body mass index, birth weight for gestational age and congenital malformations in liveborn singleton term infants born in Sweden, 1998-2019 | | | |
| --- | --- | --- | --- |
| **Comparison group** | **Infant Mortality** | **Cerebral Palsy** | **Epilepsy** |
|  | **Risk Ratio (95%CI)** ^†^ | **Risk Ratio (95%CI)** ^†^ | **Risk Ratio (95%CI)** ^†^ |
| 37 weeks vs ≥38 weeks | 1.94 (1.64,2.29) | 1.42 (1.21,1.67) | 1.20 (1.10,1.30) |
| 38 weeks vs ≥39 weeks | 1.50 (1.32,1.71) | 1.16 (1.03,1.30) | 1.12 (1.06,1.18) |
| 39 weeks vs ≥40 weeks | 1.08 (0.95,1.22) | 0.96 (0.86,1.06) | 1.01 (0.97,1.06) |
| 40 weeks vs ≥41 weeks | 0.84 (0.73,0.97) | 0.95 (0.85,1.05) | 0.96 (0.92,1.01) |
| 41 weeks vs 42 weeks | 0.78 (0.63,0.96) | 0.80 (0.68,0.94) | 0.95 (0.88,1.03) |

^†^Adjusted for maternal age, country of origin, education level, cohabitation with a partner, parity, height, smoking during pregnancy, child’s sex, year of delivery, maternal body mass index, birth weight for gestational age and major malformations.

| **Table S7.** Gestational age at birth and risk ratios (RRs) and 95% confidence intervals (CIs) for infant mortality, cerebral palsy and epilepsy, in sensitivity analysis including women with hypertensive disorders of pregnancy, singleton term infants born in Sweden, 1998-2019 | | | | |
| --- | --- | --- | --- | --- |
| **Comparison group** | **Still birth** | **Infant Mortality** | **Cerebral Palsy** | **Epilepsy** |
|  | **Risk Ratio (95% CI)** | **Risk Ratio (95% CI)** | **Risk Ratio (95% CI)** | **Risk Ratio (95% CI)** |
| 37 weeks vs ≥38 weeks | 3.83 (3.42,4.29) | 2.40 (2.05,2.80) | 1.53 (1.31,1.79) | 1.22 (1.13,1.32) |
| 38 weeks vs ≥39 weeks | 1.94 (1.75,2.15) | 1.59 (1.40,1.79) | 1.19 (1.06,1.33) | 1.11 (1.06,1.17) |
| 39 weeks vs ≥40 weeks | 0.99 (0.89,1.11) | 1.03 (0.92,1.16) | 0.95 (0.86,1.05) | 1.01 (0.97,1.05) |
| 40 weeks vs ≥41 weeks | 0.84 (0.74,0.94) | 0.78 (0.68,0.89) | 0.87 (0.78,0.96) | 0.95 (0.91,1.00) |
| 41 weeks vs 42 weeks | 0.68 (0.57,0.80) | 0.72 (0.59,0.87) | 0.80 (0.69,0.94) | 0.96 (0.89,1.03) |

^†^ Adjusted for maternal age, country of origin, education level, cohabitation with a partner, parity, height, smoking during pregnancy, child’s sex, and year of delivery.

| **Table S8.** Gestational age at birth and risk ratios for cerebral palsy (composite outcome) in term singleton infants born in Sweden, 1998-2019 | | |
| --- | --- | --- |
| **Comparison group** | **Feto-infant mortality or cerebral palsy (composite outcome)** | |
|  | Crude Risk Ratio | Adjusted Risk Ratio^†^ |
| 37 weeks vs ≥38 weeks | 2.47 (2.30,2.64) | 2.33 (2.17,2.51) |
| 38 weeks vs ≥39 weeks | 1.43 (1.36,1.52) | 1.44 (1.36,1.53) |
| 39 weeks vs ≥40 weeks | 1.05 (0.99,1.10) | 1.04 (0.98,1.09) |
| 40 weeks vs ≥41 weeks | 0.84 (0.79,0.89) | 0.85 (0.80,0.90) |
| 41 weeks vs 42 weeks | 0.77 (0.70,0.83) | 0.79 (0.72,0.86) |

^†^ Adjusted for maternal age, country of origin, education level, cohabitation with a partner, parity, height, smoking during pregnancy, child’s sex, and year of delivery.

| **Table S9.** Gestational age at birth and risk ratios for epilepsy (composite outcome) in term singleton infants born in Sweden, 1998-2019 | | |
| --- | --- | --- |
| **Comparison group** | **Feto-infant mortality or epilepsy (composite outcome)** | |
|  | Crude Risk Ratio | Adjusted Risk Ratio^†^ |
| 37 weeks vs ≥38 weeks | 1.74 (1.65,1.83) | 1.67 (1.58,1.77) |
| 38 weeks vs ≥39 weeks | 1.24 (1.19,1.29) | 1.24 (1.19,1.29) |
| 39 weeks vs ≥40 weeks | 1.02 (0.99,1.05) | 1.03 (0.99,1.06) |
| 40 weeks vs ≥41 weeks | 0.92 (0.88,0.95) | 0.92 (0.88,0.96) |
| 41 weeks vs 42 weeks | 0.88 (0.83,0.93) | 0.89 (0.84,0.94) |

^†^ Adjusted for maternal age, country of origin, education level, cohabitation with a partner, parity, height, smoking during pregnancy, child’s sex, and year of delivery.

| **Table S10.** Robustness to unmeasured confounding (E-values) of the total adjusted risk ratio expressing the relation between gestational age and infant mortality, cerebral palsy, and epilepsy, liveborn singleton term infants in Sweden, 1998–2019 | | | |
| --- | --- | --- | --- |
| **Potential mediator** | **Risk Ratio (95% CI) for outcomes** | | |
|  | **Total effect** | **E-value for RR** | **E-value for lower 95% CI** |
| **Infant Mortality** | | | |
| **37 weeks vs ≥38 week** |  |  |  |
| Asphyxia-related neonatal conditions | 2.45 (2.02,3.03) | 4.33 | 3.46 |
| **38 weeks vs ≥39 week** |  |  |  |
| Asphyxia-related neonatal conditions | 1.62 (1.31,1.82) | 2.62 | 1.95 |
| **40 weeks vs ≥41 week** |  |  |  |
| Asphyxia-related neonatal conditions | 0.82 (0.64,0.92) | 1.74 | 1.39 |
| **41 weeks vs 42 week** |  |  |  |
| Asphyxia-related neonatal conditions | 0.73 (0.59,1.00) | 2.08 | 1.0 |
| **Cerebral Palsy** | | | |
| **37 weeks vs ≥38 week** |  |  |  |
| Asphyxia-related neonatal conditions | 1.63 (1.36,2.03) | 2.64 | 2.06 |
| **38 weeks vs ≥39 week** |  |  |  |
| Asphyxia-related neonatal conditions | 1.22 (1.00,1.34) | 1.74 | 1.0 |
| **41 weeks vs 42 week** |  |  |  |
| Asphyxia-related neonatal conditions | 0.79 (0.60,0.91) | 1.85 | 1.43 |
| **Epilepsy** | | | |
| **37 weeks vs ≥38 week** |  |  |  |
| Asphyxia-related neonatal conditions | 1.24 (1.06,1.32) | 1.79 | 1.32 |
| **38 weeks vs ≥39 week** |  |  |  |
| Asphyxia-related neonatal conditions | 1.13 (1.03,1.18) | 1.51 | 1.21 |

| **Table S11.** Gestational age at birth and hazard ratios (HRs) and 95% confidence intervals (CIs) for cerebral palsy and epilepsy, singleton term infants born in Sweden, 1998-2019 | | | | |
| --- | --- | --- | --- | --- |
| **Comparison group** | **Cerebral Palsy** | | **Epilepsy** | |
|  | **Rate/10,000 child-years** | **Hazard Ratio (95%CI)** ^†^ | **Rate/10,000 child years** | **Hazard Ratio (95%CI)** ^†^ |
| 37 weeks vs ≥38 weeks | 2.07 | 1.60 (1.37,1.87) | 7.91 | 1.24 (1.15,1.35) |
| 38 weeks vs ≥39 weeks | 1.45 | 1.19 (1.06,1.33) | 7.00 | 1.12 (1.07,1.18) |
| 39 weeks vs ≥40 weeks | 1.22 | 0.95 (0.86,1.05) | 6.3 | 1.01 (0.97,1.06) |
| 40 weeks vs ≥41 weeks | 1.18 | 0.88 (0.79,0.98) | 6.1 | 0.96 (0.91,1.00) |
| 41 weeks vs 42 weeks | 1.29 | 0.81 (0.69,0.95) | 6.3 | 0.94 (0.87,1.01) |

^†^ Adjusted for maternal age, country of origin, education level, cohabitation with a partner, parity, height, smoking during pregnancy, child’s sex, and year of delivery.

| **Table S12.** Analysis of gestational age at birth and risk ratios for stillbirth, infant mortality, cerebral palsy and epilepsy according to parity (0 or ≥1) in term singleton infants born in Sweden, 1998-2019 | | | | |
| --- | --- | --- | --- | --- |
| **Comparison group** | **Stillbirth** | **Infant Mortality** | **Cerebral Palsy** | **Epilepsy** |
|  | **Risk Ratio (95%CI)** ^†^ | **Risk Ratio (95%CI)** ^†^ | **Risk Ratio (95%CI)** ^†^ | **Risk Ratio (95%CI)** ^†^ |
| **Primiparae** | | | | |
| 37 weeks vs ≥38 weeks | 3.41 (2.89,4.03) | 2.06 (1.60,2.65) | 1.16 (0.90,1.49) | 1.27 (1.13,1.43) |
| 38 weeks vs ≥39 weeks | 2.04 (1.76,2.37) | 1.54 (1.26,1.89) | 1.01 (0.85,1.21) | 1.14 (1.05,1.23) |
| 39 weeks vs ≥40 weeks | 0.97 (0.83,1.14) | 0.95 (0.79,1.15) | 0.96 (0.83,1.10) | 1.01 (0.95,1.08) |
| 40 weeks vs ≥41 weeks | 0.82 (0.69,0.96) | 0.79 (0.65,0.96) | 0.92 (0.80,1.06) | 0.94 (0.88,1.01) |
| 41 weeks vs 42 weeks | 0.60 (0.48,0.75) | 0.79 (0.61,1.04) | 0.82 (0.67,1.01) | 0.96 (0.86,1.06) |
| **Multiparae** | | | | |
| 37 weeks vs ≥38 weeks | 4.41 (3.79,5.13) | 2.81 (2.32,3.41) | 2.06 (1.69,2.51) | 1.23 (1.10,1.37) |
| 38 weeks vs ≥39 weeks | 1.89 (1.64,2.19) | 1.67 (1.43,1.95) | 1.34 (1.16,1.55) | 1.12 (1.05,1.20) |
| 39 weeks vs ≥40 weeks | 1.05 (0.90,1.23) | 1.11 (0.95,1.30) | 0.93 (0.81,1.07) | 1.01 (0.95,1.07) |
| 40 weeks vs ≥41 weeks | 0.90 (0.75,1.07) | 0.79 (0.65,0.95) | 0.84 (0.72,0.98) | 0.97 (0.91,1.03) |
| 41 weeks vs 42 weeks | 0.85 (0.63,1.13) | 0.70 (0.53,0.93) | 0.78 (0.61,1.00) | 0.91 (0.81,1.01) |
|  |  |  |  |  |

^†^Adjusted for attained age of child, maternal age, country of origin, education level, cohabitation with a partner, height, smoking during pregnancy, child’s sex and year of delivery.
